# Kansas City Veterinary College

**Published:** 1895-07

**Authors:** 


### Kansas City Veterinary College.

A complete reorganization of the Kansas City Veterinary College has taken place on a very thorough basis, in anticipation of the coming year's college work. A very distinctive feature of this change, and one to which all veterinarians will give hearty accord, is the effort to place veterinarians at the head of all the departments of instruction wherever the same could be accomplished without impairing the object of attaining a higher course of instruction. Some five of the instructors of last year, graduates in human medicine, have been replaced by some six veterinarians on the teaching staff. The following well-known veterinarians will be among the corps of instructors during the college year of 1895–96: Dr. John Forbes, M.R.C.V.S. George C. Pritchard, V.S., recently State Veterinarian of Kansas; Robert H. Harrison, D.V.S., graduate of the American Veterinary College, and the highest-honor man of his class in 1881 at that school; John Airth, M.R.C.V.S., recently appointed Inspector of the Bureau of Animal Industry; Silas S. Brooking, M.D., D.V.S. In addition to these there has been added the names of Leon Rosewald, M.D., Leo A. Schaeffer, M.D., and Nathan McVey, M.D. These additions make a corps of instructors composing the Faculty numbering eighteen, covering every subject that pertains to the teaching of veterinary science. All applicants for admission as students without college certificate or diploma, must pass an examination in accordance with the rules laid down by the Associated Veterinary Faculties of North America. A graded course of three years, not compulsory, has been planned, and is strongly urged upon the students by the Faculty.

With this added strength and many changes we feel assured that the course of instruction the coming year at this college will be better than ever, and we can only regret that the Board of Directors have not seen their way clear to establish a compulsory three years' course. The action of Pennsylvania and New York State in their new laws pertaining to future practitioners in these States should be measured at their full value, and they have set the pace for others to follow.

---

